# Early-Life Respiratory Syncytial Virus Infection, Trained Immunity and Subsequent Pulmonary Diseases

**DOI:** 10.3390/v12050505

**Published:** 2020-05-04

**Authors:** Carrie-Anne Malinczak, Nicholas W. Lukacs, Wendy Fonseca

**Affiliations:** 1 Department of Pathology, University of Michigan, Ann Arbor, MI 48109, USA; carrieam@med.umich.edu (C.-A.M.); nlukacs@med.umich.edu (N.W.L.); 2 Mary H. Weiser Food Allergy Center, University of Michigan, Ann Arbor, MI 48109, USA

**Keywords:** early-life RSV, long-term alterations, trained immunity, epigenetics, asthma

## Abstract

Respiratory syncytial virus (RSV) is often the first clinically relevant pathogen encountered in life, with nearly all children infected by two years of age. Many studies have also linked early-life severe respiratory viral infection with more pathogenic immune responses later in life that lead to pulmonary diseases like childhood asthma. This phenomenon is thought to occur through long-term immune system alterations following early-life respiratory viral infection and may include local responses such as unresolved inflammation and/or direct structural or developmental modifications within the lung. Furthermore, systemic responses that could impact the bone marrow progenitors may be a significant cause of long-term alterations, through inflammatory mediators and shifts in metabolic profiles. Among these alterations may be changes in transcriptional and epigenetic programs that drive persistent modifications throughout life, leaving the immune system poised toward pathogenic responses upon secondary insult. This review will focus on early-life severe RSV infection and long-term alterations. Understanding these mechanisms will not only lead to better treatment options to limit initial RSV infection severity but also protect against the development of childhood asthma linked to severe respiratory viral infections.

## 1. Introduction

Studies have shown that up to 68% of infants may be infected with respiratory syncytial virus (RSV) in their first year of life, and nearly all children will be infected by two years of age [1]. RSV infection is the leading cause of childhood hospitalization [2,3]. In healthy infants, most infections result in mild disease, but children under six months of age during peak RSV season are most at risk of developing severe RSV disease [4,5], which increases the possibility of developing childhood asthma and recurrent wheezing later in life [2,3]. RSV-related death is a significant cause of mortality in developing countries and individuals with pre-existing conditions. It is believed to be the second most likely cause of death by a single pathogen in children under one year of age, worldwide [1,6]. In addition to a severe primary infection, RSV re-infection is common throughout life, and up to 75% of infants infected by 12 months of age will become re-infected before they reach the age of two. Symptoms of the disease may wane with re-infection; however, the elderly, immunocompromised individuals, and those with chronic conditions, including asthma, are at high risk of developing lower respiratory tract infections making RSV a significant cause of morbidity and mortality in these individuals [1,6].

There is currently no vaccine available or a sufficient option for antiviral therapy, partially due to our incomplete understanding of how severe immunopathology is developed. In the 1960s, a vaccine trial was conducted with suboptimal results; vaccinated infants became severely exacerbated upon exposure to live virus leading to severe disease as well as two infant deaths [7,8,9]. There is, however, a monoclonal antibody (palivizumab) that does show success when given to high-risk infants prophylactically, by diminishing RSV immunopathology, but not infection, and leading to decreased hospitalization compared to control groups [10]. Another available immunotherapy is motavizumab, which is an RSV-specific monoclonal antibody with a significantly higher in vitro binding affinity for the RSV fusion protein than palivizumab [11] and similar safety profiles as palivizumab in children [12]. Observational studies and recent randomized controlled trials demonstrate that preventing severe RSV disease decreases recurrent wheezing and 1-year wheezing outcomes [13,14]. In contrast, a retrospective cohort study showed that RSV immune prophylaxis was not associated with reduced asthma [15]. However, at least half of all children that are hospitalized due to severe RSV are previously healthy individuals [1] and would not qualify for prophylactic treatment. Therefore, identifying mechanisms leading to disease and targeting new treatment options for the general population is a critical unmet need.

Along with the initial disease complications associated with primary RSV infection, RSV-driven Th2-immunity skewing has also been implicated in leading to airway restructuring linked to exacerbated allergic responses later in life in animal models [16]. Up to 48% of infants who were hospitalized for severe RSV-associated bronchiolitis and/or lower respiratory tract infection go on to develop asthma during their childhood [17,18]. Asthma is the most common chronic childhood illness, affecting approximately 5 million children under 18 years of age, including 1.3 million children under five [19]. During childhood, males have higher occurrences of asthma and wheezing than females at a 2:1 ratio [20]. Additionally, males are approximately twice as likely to become hospitalized than females due to severe RSV infection [21]. Thus, the risk for severe RSV infection and RSV-associated asthma development is higher in males than in females [22].

In this review, we will summarize several clinical and experimental studies related to severe RSV disease and subsequent risk for asthma and allergic responses later in life. The use of different experimental approaches in human and animal models have been crucial to understanding the role of RSV on chronic respiratory diseases. It is important to note that detailed mechanistic studies, especially those related to neonatal RSV infection, need to be conducted using animal models and/or in vitro cell systems due to necessary limitations on clinical testing and sample collection in human subjects. We will discuss how early-life RSV infection alters the immune response and may have long-term or systemic effects that can lead to severe complications throughout life. 

## 2. Early-Life RSV Immunopathology

RSV is one of the most frequent viral causes of lower respiratory tract infection in children and is implicated in up to 80% of all bronchiolitis cases [1,21]. Bronchiolitis is the cause of nearly 20% of infant hospitalization in the United States, indicating that RSV is a significant health care burden, even in industrialized countries [1]. It has been reported that infants under six months of age are the most susceptible group to develop severe RSV disease, suggesting that the age of infection plays a critical role in RSV pathology [4]. Other reported specific factors linked with RSV disease severity are viral load, induction of interferon (IFN), and exacerbated inflammation directed by innate immune cells and overexpression of neutrophil-related genes [23,24].

RSV predominantly infects ciliated epithelial cells in the airways but can also lead to infection in the subepithelium and neighboring immune cells, such as macrophages and dendritic cells [25,26]. The major histopathologic characteristics of RSV infection include acute bronchiolitis, mucosal swelling of the airways, and airway obstruction caused by sloughed epithelial cells and mucus production [1,26,27]. Numerous studies in humans and mice have shown that RSV plays a role in reducing innate cytokine production that is necessary for appropriate antiviral responses [23,24,28,29,30,31]. Both viral replication, as well as RSV-associated immunopathology, can lead to RSV disease symptoms. Although the underlying mechanisms related to these latter responses are not clear, they are supported by clinical data and may be successfully treated by identifying the immune environment. 

Initial RSV response is driven by innate cytokines, such as IL-25, IL-33, and thymic stromal lymphopoietin (TSLP) that are released from the airway epithelial cells following infection [32,33,34,35]. These cytokines (a.k.a. Alarmins) have the ability to activate the immune response through interactions with dendritic cells, T cells, and innate lymphoid cells [35,36,37,38,39]. During a typical viral infection, the immune response is dictated by dendritic cells (DC) that activate the immune system and instruct T cells toward distinct T helper-type responses [40]. An in vitro study has shown that RSV can skew the immune response towards a Th2-type response by inhibiting the production of type 1 IFN and subsequently decreasing the Th1 antiviral response [41]. This lack of an antiviral response, as well as skewing towards dysregulated Th2/Th17, has been correlated with severe disease in infants, mice and in vitro modeling [28,29,42,43]. Furthermore, type I IFN pathways are associated with Th1 immune responses and decreased hyper-reactivity after RSV re-infection in mice [44]. RSV is a poor inducer of IFN, and it has been described that infants and neonatal mice cannot induce a robust type I IFN immune response [24,45,46]. Additionally, studies with neonatal mouse RSV infection have demonstrated that there are persistent changes in the lung associated with unresolved immunopathology, that include increased mucus production, increased inflammation, and persistent expression of inflammatory cytokines, including IL-33 and TSLP [34,46,47,48,49]. 

A nonpathogenic response requires a balance of antiviral and regulatory immune cells, and inappropriate immune responses have been linked to the development of pathologic responses following RSV infection in mice as well as using in vitro modeling in human cell lines [28,29,42]. For example, the Th2 and Th17 type immune responses have been implicated in severe RSV disease as well as the development of many lung pathologies, such as allergy and asthma [18,28,29]. The Th2 and Th17 responses promote the infiltration of eosinophils and neutrophils into the lungs of mice leading to an inflammatory-allergic cellular environment which dampens effector functions, such as the secretion of IFN-γ [28,50]. As a result, clearance of RSV is delayed, and instead, immunopathology is promoted. Th2 cytokine production (IL-4, IL-5, and IL-13) leads to antibody class switching to IgE, eosinophil recruitment, and mucin production, characteristics of both severe RSV disease as well as asthma that can lead to lung dysfunction [18,28]. In patients and experimental animal models with these airway diseases, including RSV, increased numbers of goblet cells (mucus-producing cells) replace the bronchial epithelium of the lung [51]. Goblet cell metaplasia in airway diseases is accompanied by significant airway inflammation; therefore, inflammatory cells and their secreted mediators are believed to directly act on airway progenitor cells to induce goblet cell formation [51,52,53]. 

A significant producer of Th2 cytokines are the innate lymphoid 2 cells (ILC2), known to be involved in lung disease pathogenesis. ILC2 are a relatively rare population of innate cells of lymphoid origin, but unlike T cells or B cells do not contain an antigen receptor, and are therefore nonspecific innate responding cells [54]. It has been suggested that ILC2 are far more potent than CD4+ T cells in their induction of type-2 cytokines; in fact, it is estimated that ILC2 produce 10 times more cytokines than T cells on a per cell basis [32]. Innate cytokines, including TSLP, IL-25 and IL-33, which are secreted following infection of murine and human airway epithelial cells by RSV [34,35], are known inducers of ILC2 differentiation and activation [36,37,55]. ILC2 numbers are increased in RSV-infected murine lungs to produce IL-5 and IL-13, cytokines that promote the development of airway inflammation and mucus production as described above [47,56]. Using a mouse model, Stier et al. linked the early induction of effector ILC2 to the development of airway hyperreactivity and mucus production associated with RSV through TSLP-driven induction of IL-13 producing ILC2 [32]. Age-related IL-33 production in mice was shown to be necessary to induce ILC2 and lead to Th2-driven immunopathology following neonatal RSV infection [47]. In addition to the RSV-driven immunopathology, the IL-33/ILC2 mechanism has also been implicated in pathologies related to early-life rhinovirus infection [57]. It has been shown that RSV infection in mice promotes ILC2/IL-13-driven Th2 response through the activation of the uric acid pathway and the induction of innate cytokines (IL-1β, CCL-2, TSLP, and IL-33) [34]. Moreover, the study also showed that the treatment of IL-33 during RSV infection diminished the expression of IFN-β in murine airway epithelial cells, suggesting novel control of type 1 IFN during RSV infection [34]. Importantly, this study correlated with human data showing that uric acid is also increased in bronchiolar lavage fluid from RSV-infected infants [34]. The inhibition of uric acid production or the downstream-induced IL-1 abrogated the severe RSV response, suggesting a possible clinical treatment option [34].

Other factors that can affect RSV immunopathology and the severity of the disease are genetic polymorphisms [58,59,60,61,62]. In a prospective study, infants with severe bronchiolitis that were hospitalized three times over a 2-year period were evaluated for single nucleotide polymorphisms ( SNPs) in immune response genes and compared with healthy controls [58]. The authors observed that the SNP rs2107538*CCL5 was associated with bronchiolitis caused by respiratory syncytial virus (RSV) and RSV-subtype-A while the SNP rs1060826*NOS2 was associated with bronchiolitis caused by rhinovirus [58], indicating viral-specific polymorphisms. Furthermore, SNPs in Toll-like receptors (TLRs), including rs4986790*TLR4, rs1898830*TLR2, rs7656411*TLR2, rs352162*TLR9, and rs187084*TLR9 as well as rs2280788*CCL5 were associated with severity of bronchiolitis [58,59]. TLR4 has been described to be involved in the innate immune response to RSV by recognition of RSV F glycoprotein. TLR4 is activated during RSV bronchiolitis and genetic variations of *TLR4* (Asp299Gly and Thr399Ile mutations) represent risk factors for RSV infection [60,61]. Additionally, other studies found that severe RSV bronchiolitis is associated with SNPs in *TLR4* (rs4986790 and rs4986791) [60,62].

## 3. Early-Life RSV and Long-Term Lung Alterations

In addition to severe disease following initial RSV infection, many studies have indicated that the immune system is persistently altered following this early-life infection, which may impact future immune responses later in life [47,48,56,63,64]. A clinical study that compared disease severity with hospitalization rate showed that children with mild RSV disease had higher levels of type I IFN and decreased inflammatory genes when compared to children with severe disease [24]. These data correlated increased expression of IFN with decreased odds of hospitalization [24], demonstrating the importance of IFN in the immunomodulation of RSV pathology. It has also been observed that children hospitalized with severe RSV infection maintained the immune profiles after 1 month of the infection [23]. 

Studies with murine neonatal RSV infection have demonstrated that there are persistent changes in the lung that include mucus production and increased immune cell populations that persist in the lung, including ILC2 [47,48,49,56]. Furthermore, studies from our laboratory show a direct correlation between early-life RSV infection and the enhanced development of allergic disease later in life. Importantly, these responses are more severe in male mice and correlate with clinical data showing that males are more susceptible both to severe RSV as well as enhanced allergic disease [48]. These studies determined an increased presence of inflammatory immune cells, such as DCs, macrophages, and ILC2 for several weeks postinfection as well as type 2 and innate cytokines [48] that drive chronic disease [32,33,34,48]. The use of TSLPR knockout (TSLPR-/-) mice abrogated this enhanced allergic response through a decreased in ILC2 and Th2 cytokine production, indicating a direct role for TSLP in this model [48]. These data correlate with studies from the Ziegler lab that identified TSLP during early-life RSV infection in mice as a key driver of enhanced RSV disease upon secondary infection later in life [65]. Importantly, these studies suggest TSLP as a potential clinical target, and current clinical trial testing is ongoing in adult asthmatics using a monoclonal anti-TSLP antibody [66,67].

The detrimental role of increased ILC2 following RSV infection may be two-fold—1) as an inducer of pathogenic inflammation as described above and 2) in altering the structural/developmental process of the lung in neonates. The early-life lung in both humans and mice is predisposed to a type 2 immune environment for proper lung development to occur. This predisposition may enhance the detrimental effects of RSV by hijacking these programs, leading to severe immunopathology as well as improper lung development. IL-33-specific ILC2 have a known role for normal lung development [68,69] and it has been documented that an influx of ILC2 into the mouse lung at 7 days of age leads to increased IL-13 production for proper alveolarization [68]. Furthermore, the Lambrecht lab determined that it is during this timeframe that young mice are most susceptible to allergic airway disease due to this heightened type-2 cell milieu [69]. Studies have also determined that during this time of early life, the extracellular matrix is being established through the expression of elastin and collagen for normal lung development in mice [70]. However, re-activation and/or overactivation of these programs have been linked to severe lung dysfunction, including fibrosis [71,72]. ILC2 have also been implicated in the involvement of detrimental remodeling of the mouse lung along with the innate cytokines, IL-33 and TSLP [73,74,75,76,77]. Airway remodeling, including dysregulated extracellular matrix deposition, is a hallmark symptom of asthma [78] and RSV infection has previously been suggested to lead to remodeling with collagen deposition [16]. ILC2 production of amphiregulin, while necessary for lung development and repair following epithelial injury, may lead to lung fibrosis [73,74,75]. Furthermore, mouse studies have indicated that TSLP is required for the initiation/persistence of airway remodeling during chronic allergy [76]. These studies suggest that persistent IL-33, TSLP, and activation of ILC2 following RSV infection may lead to alterations in inflammatory responses as well as promote altered lung function as a consequence of tissue restructuring and possible impairment of lung development.

**Figure 1:**
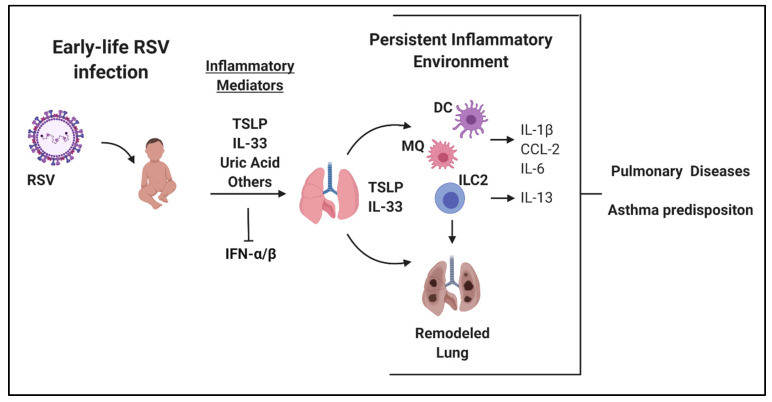
Overview of early-life respiratory syncytial virus (RSV) infection and long-term local alterations within the lung. Figure created using Biorender.com.

These studies highlight the role that the innate immune system has on viral immunity and possible long-term consequences on lung function and environment. These include alterations in not only innate immune cells themselves, such as myeloid cells (DCs, macrophages, etc.) and ILC2 but also the cytokine milieu that they express, including increased expression of IL-33 and TSLP as well as IL-13 with concomitant inhibition of type-1 IFN. Thus, innate immune responses during early-life RSV infection appear to be central to the development of long-term lung environment as well as structural alterations within the lung (Figure 1). 

## 4. Trained Innate Immunity Following Early-Life RSV Infection

Trained immunity involves innate cells such as myeloid cells (including dendritic cells), natural killer (NK) cells, and innate lymphoid cells (ILCs) and their interactions with pattern recognition receptors (PRRs) and effector cytokines. In contrast to classical adaptive memory, the increased responsiveness to secondary stimuli during trained immunity is not specific for a particular pathogen and it is mediated through signals involving transcription factors, epigenetic reprogramming and metabolic alterations [79,80,81]. A distinguishing feature of trained innate immunity is the ability to mount a qualitatively different and often much stronger transcriptional response when challenged with pathogens or danger signals. In myeloid cells, many loci encoding inflammatory genes are in a repressed configuration [82,83]. Upon primary stimulation, massive changes are observed in these loci with alterations driven by the recruitment of transcription factors to enhancers and gene promoters [79]. Transcription factors then recruit coactivators that locally modify chromatin to make it more accessible to transcriptional machinery [82,83]. Maintenance of this enhanced accessibility may lead to more efficient induction of genes primed by the initial stimulation [84]. The persistence of histone modifications deposited at promoters or enhancers after the initial stimulus may impact the secondary response [79,85]. Therefore, the observed long-term persistence of some histone modifications in myeloid cells after removal of the initial activation stimulus may reflect either stability of these marks or the sustained activation of the upstream signaling pathways and transcription factors that control their deposition. It is reasonable to ascertain that the innate immune system, specifically myeloid populations such as macrophages and DCs, may be persistently “trained” following an early-life RSV infection that leads to an inappropriate response to unrelated exposures, such as those observed following a future allergen challenge.

### 4.1. Myeloid Cell Modification Following RSV Infection

Many studies have shown that myeloid cells are significantly altered following viral and bacterial infections [79,85,86,87,88]. Importantly, clinical data indicate that myeloid cells, such as dendritic cells, are key players in the immunopathology associated with RSV infection in infants [25,89,90] which supports further investigation of these cell populations. Dendritic cells (DCs) play a crucial role in the development of the immune response and link innate and adaptive immunity by instructing T cells toward a Th1 (antiviral), Th2 (antiparasitic or allergy-associated) or Th17 (antibacterial or autoimmunity) type response. During a typical viral response, the DCs instruct T cells toward antiviral Th1 via the production of type-1 interferon (IFN), which leads to the production of IFN-γ and proper viral clearance [91]. However, RSV has the ability to dampen type-1 immunity through multiple mechanisms, including direct inhibition of type-1 interferon and antigen decoys to evade the immune system [92,93,94]. In the absence of type-1 immunity, the DCs produce cytokines and chemokines that skew the response towards Th2 [50,92,95]. 

It has been shown that the numbers of DCs increase in the lung following RSV infection in both humans and mice [25,95,96,97]. Analysis of nasal washes collected from children during an RSV infection determined that DCs were increased in the lung and furthermore, levels of DCs in the blood were decreased, correlating with recruitment to sites of infection [96,97]. However, it is important to note that different DC subsets (plasmacytoid DC (pDC) and conventional DC (cDC)) have been shown to play differential roles in the RSV immune response. The pDC population has been demonstrated in animal models as well as human studies to have a protective role through the production of IFN-α for viral clearance as well as regulation of the Th2 response, limiting airway hyperreactivity and mucus production [25,46,98,99], whereas high levels of cDCs correlate with severe RSV disease during infant hospitalization [99]. Studies in neonatal mouse models of RSV re-infection demonstrated that RSV-infected neonates produced significantly lower levels of type I IFN along with reduced pDC recruitment in the lungs that led to increased Th2 response and significant pathology, indicating a protective role for the pDC subset which is lacking in neonates [46]. In a separate mouse study, the role of IFN-α administration prior to neonatal RSV infection was examined and led to decreased Th2-biased immunopathogenesis during reinfection with downregulation of IL-4Rα [100]. Furthermore, it has been shown that RSV impacts the expression of antiviral IFN-stimulated genes (ISGs) by human monocyte-derived DCs [101]. Similar effects were reported in infected mouse lung epithelial cells whereby RSV decreased the expression of ISG-15, whereas RSV mutant virus lacking the structural G protein did not alter ISG-15 [102]. 

On the other hand, cDCs have been implicated in promoting the Th2 response by upregulation of CCR6 and CCL20 secretion leading to the recruitment of Th2 cells to the lung [25,103]. Interestingly, correlations have been shown between the absolute number of cDCs and increased levels of inflammatory cytokines (i.e., IL-6, CCL-2) in humans supporting cDCs as a source of inflammation within the nasal cavity during RSV infection [25,96,97]. Simply increasing numbers of cDCs within the mouse lung can lead to a pathogenic response during RSV infection [103]. Furthermore, multiple mouse studies have indicated that RSV infection leads to enhanced numbers of cDCs infiltrating into the lungs and treatments that limit RSV disease severity also led to a decreased number of cDCs [34,48,104], suggesting these cells play a role in enhanced disease.

Many mouse and human studies have also identified expression of several DC-linked chemokines (CCL-2, CCL-3, CCL-5, CxCL-1, CxCL-2) that contribute to the pathogenesis of asthma, RSV-induced exacerbation, and the clearance and resolution of the ongoing pathology [92,103,105]. 

In addition, chemokine receptors (CCR6, CCR7) have been identified that regulate the recruitment of specific immune cells involved in the response to RSV and/or allergen-induced asthma-like responses [103,106,107]. Furthermore, RSV predisposes animals to more severe allergen responses [107,108] and the most prominent cell that appears to be involved in chronic disease that is enhanced/exacerbated by RSV is the inflammatory/myeloid dendritic cell (mDC). It has previously been identified that RSV-exposed mDCs are sufficient to promote a more severe and accelerated disease environment in mice when exposed to allergens [108]. Understanding how RSV infection influences DC function to alter the immune environment is critical to understanding how these responses impact pulmonary pathogenesis, leading to exacerbations later in life. It is likely that RSV and other viral infections, including rhinovirus infection, uncover an underlying phenotype and reinforce the immune environment by further altering pathogenic immune responses, possibly through metabolic and epigenetic modifications consistent with trained immunity as described above. 

### 4.2. Metabolic Modification Following RSV Infection

The concept that our immune responses are influenced by environmental factors including microorganisms, pollution, diet and pathogen exposure that lead to shifts in microbiome and metabolic profiles, is important for understanding disease progression. Interestingly, a study of infants hospitalized due to bronchiolitis caused by RSV or rhinovirus (RV) infection determined that the microbiome and metabolic programming varied significantly between infants hospitalized due to RSV vs. RV [109]. RSV was associated with metabolites from a range of pathways, including carbohydrate, lipid, amino acid, and energy metabolism with a microbiome dominated by *Streptococcus pneumoniae* while RV infection was associated with increased levels of essential and nonessential N-acetyl amino acids and high relative abundance of *Haemophilus influenzae* [109]. These studies suggest that large metabolic and metagenomic shifts occur following pathogen exposure but that these shifts are dependent upon and possibly exclusive to the specific pathogen encountered. Studies of RSV using animal models have begun to further characterize these mechanisms. Factors affecting the microbiome and RSV have been previously reviewed [110], therefore, the following studies will focus on metabolic alterations. 

Untargeted metabolic analysis of human airway epithelial cells during RSV infection has shown that several metabolites are altered after infection, with upregulation of nucleotides, amino acids, amino and nucleotide sugars, and metabolites of the central carbon pathway while the metabolism of lipids were downregulated [111]. The authors also observed increased levels of oxidized glutathione and polyamines, that were associated with oxidative stress [111]. It has also been shown that RSV infection of human epithelial cells alters antioxidant responses by increasing the degradation of the transcription factor NRF2, which controls the expression of several antioxidant enzymes, such as catalase [112]. Furthermore, in a separate study, intranasal treatment with polyethylene glycol-conjugated catalase (PG-CAT) in RSV-infected mice showed a reduction of H_2_O_2_ in the airways and was protective against RSV-induced pathology [113], suggesting that metabolic regulation during RSV infection is essential to determine the outcome of the disease.

Metabolic reprogramming of macrophages and dendritic cells following pathogen exposure have been identified in the literature [87,114,115,116,117]. For example, activation of macrophages and DCs by pro-inflammatory stimuli causes them to undergo a metabolic switch towards glycolysis and away from oxidative phosphorylation [115], which has been further characterized in DCs during RSV infection in mice that leads to Th2 immunopathology [117]. Additionally, the transcription factor hypoxia-inducible factor-1α (HIF-1α), upregulated following RSV infection [118,119] has been shown to play an important role in the pro-inflammatory response in both macrophages and DCs [115]. Fonseca et al. showed that supplementation of mice with *Lactobacillus johnsonni* (LJ) prior to RSV infection led to significant alterations of the bone marrow-derived dendritic cells (BMDCs) upon ex vivo analysis [114]. These changes include shifts in antigen presenting cell (APC) markers and modified innate cytokine production which together led to decreased immunopathology and Th2 skewing through alterations in the metabolic profile, specifically in regards to short-chain fatty acids, such as Docosahexaenoic acid (DHA), shown to be protective during RSV challenge [114]. Additional mouse studies recently showed that RSV infection leads to upregulation of the uric acid pathway that leads to enhanced disease, partially through IL-1β expression in bone marrow-derived macrophages [34]. Together, these studies determine a link between metabolic alterations of myeloid cell populations within the bone marrow following RSV infection and the development of severe immunopathology.

### 4.3. Dendritic Cell Epigenetic Modification Following RSV Infection

Epigenetic modification of immune cells will be critical to understand both innate and acquired immune responses. Mouse studies have shown that the regulation of DC function can be modified by epigenetic changes to chromatin structure during maturation with CCR7 [120] as well as during severe septic disease regulating IL-12 [121]. Epigenetic modifications are also known to occur in DCs during the inflammatory response to RSV. KDM5B, an H3K4 demethylase, is upregulated following RSV infection of murine and human DCs [30]. Since H3K4me3 is an activating marker, this demethylase acts as a transcriptional repressor to specific gene targets, especially Type I IFN, leading to reduced IFN-γ and increased type 2 immunity [30]. Another key epigenetic gene modifier is the repressive H3K27 trimethylation and removal of this mark by KDM6 demethylases is associated with gene activation. Studies from our lab have shown that the KDM6 family of enzymes are crucial for immunopathology following RSV infection in mice through alteration of cytokines and antigen presenting cell function leading to type 2 immunity [104]. These data correlate with studies by Doñas et al. that showed that inhibition of KDM6a/b enzymatic activity leads to decreased inflammation during murine experimental autoimmune encephalitis by creating a tolerogenic DC phenotype [122]. Interestingly, while both KDM5B and KDM6 are upregulated in BMDCs following RSV infection, they do not appear to regulate the same genes and importantly differentially impact T cell responsiveness. Thus, there appears to be an epigenetic program that controls multiple chromatin-modifying enzymes that alters the ability of DCs to function appropriately during RSV infection. 

**Figure 2:**
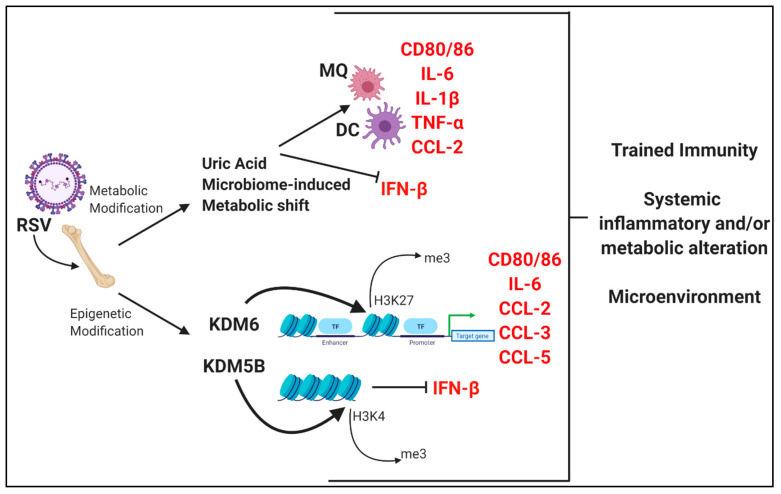
Overview of systemic alterations within the bone marrow following early-life RSV infection. Figure created using Biorender.com.

These data presented here along with studies describing the trained immunity phenomenon suggest that RSV infection is capable of altering the immune system in such a way that may lead to trained immunity in systemic populations within the bone marrow. These modifications are found at both the metabolic level as well as through epigenetic/transcriptional alterations (Figure 2) which together may persistently alter genes in such a manner that upon secondary exposures leads to detrimental effects.

## 5. Conclusions

RSV continues to be a significant healthcare burden [1,4,5,6] as well as a significant causative agent for the development of childhood asthma [2,3]. While the field has made striking discoveries in RSV biology and mechanisms, additional studies to define specific mediators and mechanisms are needed. Alteration of DCs and ILC2 during RSV infection may be central in not only initial RSV disease severity but also persistent alterations found within the lung and bone marrow. The immune system may essentially “lock” into an early-life type 2 phenotype, affecting lung function, structure, and development, as well as future inflammatory responses. 

The studies described have elucidated novel and exciting aspects of RSV-driven immunopathology and suggest that it is imperative to consider regulating the innate immune system during RSV disease. Importantly, these studies have uncovered many pathways that may potentially be targeted and ultimately lead to better treatment options (Table 1). Furthermore, the development of a successful vaccine candidate will be informed by these studies which will decrease RSV disease and limit the development of childhood asthma.

## Figures and Tables

**Table 1:** Overview of Current and Predicted RSV Therapeutic Targets.

Target	Clinical Status	Advantages	Limitations
RSV Fusion Protein	Monoclonal antibodies: Approved: Palivizumab. Phase 3 clinical trials in children: Motavizumab (source: clinicaltrials.gov)	Direct RSV-specific target	Currently only routinely administered to high-risk patients
IL-33	Monoclonal antibody: SAR440340 (REGN3500) in Phase 2 clinical trial (source: clinicaltrials.gov)	Studies suggest safety and efficacy for control of asthma and pulmonary dysfunction compared to placebo	Not yet tested in pediatric population No clinical studies showing results in RSV patients No improvement over dupilumab for asthma treatment
TSLP	Monoclonal antibody in clinical trials: Tezepelumab: Phase 3 in adults and adolescents (source: clinicaltrials.gov)	Safety and efficacy for severe asthma and viral-induced asthma exacerbations [66,67]	Not yet tested in pediatric population No clinical studies showing results in RSV patients
IL-4Rα	Monoclonal Antibody Approved: Dupilumab	Targets both IL4 and IL13 and clinical trials showed reduction in Th2 responses [123]	Only approved in adults and children older than 12. Also not approved for acute diseases
IL-1β	IL-1 receptor antagonist: Anakinra	Approved for use in pediatric population	Not yet tested for RSV-specific disease in humans
Uric Acid Pathway	Xanthine oxidase inhibitor: Allopurinol	Approved for use in pediatric population	Not yet tested for RSV-specific disease in humans
KDM5/KDM6	No specific histone demethylase KDM5 or KDM6 inhibitors are currently in clinical trials	Targets the overall inflammatory response and may be useful during the later stages of disease or to induce resolution later in life	Non-specific targeting will likely lead to many off-target side effects
IFNα/β	Numerous recombinant protein IFNα/β drugs are already approved and widely used	Enhancement of type-1 IFN response may clear virus faster as well as reduce the risk for immunopathology Approved for use in pediatric population	May need to be administered early during RSV infection Risk of developing enhanced Th1 disease (i.e., autoimmunity) and/or ISG-driven cytokine storm
